# Barriers and Facilitators for Health Behavior Change among Adults from Multi-Problem Households: A Qualitative Study

**DOI:** 10.3390/ijerph14101229

**Published:** 2017-10-15

**Authors:** Gera E. Nagelhout, Lette Hogeling, Renate Spruijt, Nathalie Postma, Hein de Vries

**Affiliations:** 1 Department of Health Promotion, Maastricht University (CAPHRI), Maastricht 6200 MD, The Netherlands; hein.devries@maastrichtuniversity.nl; 2 Department of Family Medicine, Maastricht University (CAPHRI), Maastricht 6200 MD, The Netherlands; 3 IVO Addiction Research Institute, Rotterdam 3021 DM, The Netherlands; 4 Chair Group Health & Society, Department of Social Sciences, Wageningen University, Wageningen 6700 EW, The Netherlands; lette.hogeling@wur.nl; 5 Stimenz, Apeldoorn 7311 JD, The Netherlands; R.Spruijt@stimenz.nl (R.S.); nathaliepostma@LIVE.NL (N.P.)

**Keywords:** healthy lifestyle, multi-problem households, smoking, physical activity, nutrition, alcohol, social class, Netherlands

## Abstract

Multi-problem households are households with problems on more than one of the following core problem areas: socio-economic problems, psycho-social problems, and problems related to child care. The aim of this study was to examine barriers and facilitators for health behavior change among adults from multi-problem households, as well as to identify ideas for a health promotion program. A qualitative study involving 25 semi-structured interviews was conducted among Dutch adults who received intensive family home care for multi-problem households. Results were discussed with eight social workers in a focus group interview. Data were analyzed using the Framework Method. The results revealed that the main reason for not engaging in sports were the costs. Physical activity was facilitated by physically active (transport to) work and by dog ownership. Respondents who received a food bank package reported this as a barrier for healthy eating. Those with medical conditions such as diabetes indicated that this motivated them to eat healthily. Smokers and former smokers reported that stress was a major barrier for quitting smoking but that medical conditions could motivate them to quit smoking. A reported reason for not using alcohol was having difficult past experiences such as violence and abuse by alcoholics. Mentioned intervention ideas were: something social, an outdoor sports event, cooking classes, a walking group, and children’s activities in nature. Free or cheap activities that include social interaction and reduce stress are in line with the identified barriers and facilitators. Besides these activities, it may be important to influence the target group’s environment by educating social workers and ensuring healthier food bank packages.

## 1. Introduction

Multi-problem households are households with problems on more than one of the following core problem areas: socio-economic problems (e.g., financial debts, unemployment), psycho-social problems (e.g., domestic violence, psychiatric disorders), and problems related to child care (e.g., neglect or maltreatment) [1]. These problems are often chronic, complex, and intertwined, they exist from generation to generation, and they cause high levels of psychological distress for those involved [1,2,3]. Multi-problem households can be found in any social, cultural and economic group [4]. Because of the nature of the most common problems in these households (financial problems, unemployment, unstable housing) and the screening for these households as part of social work policies (low income, unemployment benefits, low education) [5,6], multi-problem households with a low socio-economic status are the group that is most often seen by social workers [3].

Adverse family conditions and low socio-economic status are known predictors for unhealthy lifestyle behaviors such as physical inactivity, unhealthy nutrition, smoking, and alcohol abuse [7,8,9,10,11]. A number of barriers for health behavior change among low socio-economic status groups have been identified in previous research. High levels of psychological distress [9,10,12,13,14], less concern with the future consequences of health decisions [9,14], a lack of knowledge about health risks and about healthy behaviors [8,14], and lower self-efficacy and self-control to change health behavior [9,12,14] are among the psychological barriers for health behavior change among low socio-economic status groups. Additionally, these groups have been found to use less effective strategies to improve health such as skipping meals and using less effective smoking cessation aids [8,14], they have social networks of friends and family with unhealthy lifestyles [9,12,14], they live in neighborhoods with more fast-food restaurants, liquor stores, tobacco shops, more marketing of unhealthy products, and less public parks [9,10,14], and the costs of a gym membership, walking shoes, fruits and vegetables, or smoking cessation aids is an important barrier for them [8,9,10,14,15].

Whereas the above mentioned studies have examined low socio-economic status groups, few studies have examined health behaviors among the specific group of multi-problem households [2,5,16]. In The Netherlands, about 1.5% of the households are multi-problem households [1]. This is a smaller group than those in The Netherlands with a low socio-economic status: 7.6% of the Dutch households have less budget than is considered an acceptable minimum standard of living [17] and 13.4% of the population aged 25 years and older has only completed primary education [18]. Despite the small size of the group of multi-problem households, it is an important target group for health promotion because of a clustering of unhealthy behaviors in this group, combined with high levels of psychological distress. Additionally, the fact that this group often gets support from social workers can be seen as an opportunity to also address health behaviors. However, Dutch social workers of multi-problem households mostly focus on helping families with their most pressing problems, which are often related to financial problems and the safety of the children, rather than stimulating health behavior change. Therefore, developing targeted health promotion interventions for multi-problem families is needed. In order to inform intervention development, it is important to know what the specific barriers and facilitators are for health behavior change among this group [19]; yet this information is not available.

The aim of the current study was to identify barriers and facilitators for health behavior change regarding physical activity, nutrition, smoking, and alcohol use among adults from multi-problem households in The Netherlands, as well as to identify ideas for program development. Another goal of this study was to examine similarities and differences in perspectives of adults from multi-problem households and social workers who work with this group.

## 2. Methods

### 2.1. Design

A qualitative study was performed between June and December 2016 involving semi-structured interviews with 25 adults from multi-problem households (hereafter called ‘participants’) living in the municipality of Apeldoorn in The Netherlands (a municipality with 160,000 inhabitants). A qualitative study design was chosen to gain in-depth understanding about barriers and facilitators of this specific study group without having to give predefined response options.

After analyzing the data of the interviews, a focus group interview was performed in February 2017 with eight of the social workers who helped with the recruitment of participants. During the focus group interview, the main findings from the interviews with participants were discussed and social workers were asked to reflect on these results. 

### 2.2. Sample

Eligibility criteria for participants were: 18 years or older, receiving intensive family home care for multi-problem households or recently stopped receiving this care, and living in the municipality of Apeldoorn. Participants who were in the middle of an urgent crisis situation (e.g., safety of the children at risk or an upcoming housing eviction) were not recruited for the study based on recommendations by social workers, who expected that these participants would probably be too preoccupied with their crisis situation to concentrate on an interview about health behaviors. Participants were purposively selected to ensure variation in gender, age, and age of the children. Participants were recruited to take part in the interview by their social worker either during a face-to-face meeting or by telephone. Social workers assessed eligibility, gave potential participants a flyer about the study, and asked them to participate. Participants signed an informed consent form before taking part in the interview. They were reimbursed with a gift voucher of 5 Euro and a smart watch was raffled among the participants. The eligibility criterion for social workers was that they provide intensive family home care or welfare support to multi-problem households as part of a team of the municipality of Apeldoorn. They were recruited to take part in the focus group interview by email invitation. Eight of the eighteen invited social workers participated.

### 2.3. Data Collection

Interviews with participants were conducted at their homes by the first author, who is trained in qualitative interviewing. During eight of the interviews it was not possible to conduct the interview with no one else present in the same room (e.g., children, partner, or social worker). Most participants lived in small houses or apartments, and two participants lived in a trailer. Interviews with participants lasted on average 31 minutes (ranging between 16 and 46 minutes).

The interview guide is shown in Table 1. The interview covered the four health behaviors (physical activity, nutrition, smoking, and alcohol use), asking participants first about their current health behaviors (e.g., ‘what kind of physical activity do you perform?’ or ‘do you smoke tobacco?’) and their intentions for behavior change (e.g., ‘do you want to do more physical activity?’ or ‘do you want to quit smoking?’). These were more quantitative questions that served the purpose of getting some background information about the participant and asking some easy questions first before asking more in-depth qualitative questions. To assess barriers, participants who wanted to change their behavior but had not done so, were asked what made it hard or what was stopping them. To assess facilitators, participants who had changed their behavior were asked how they did it and what helped them. Failed attempts at behavior change were also discussed, both to assess barriers (what made them fail) and to assess facilitators (what made them succeed initially). Participants who answered very shortly on these questions about barriers and facilitators were asked to elaborate more about this. After talking about the barriers and facilitators, the interviewer explained about the health promotion intervention that will be developed and asked whether participants had ideas for what such a program could consist of. Each participant also completed a short self-administered questionnaire assessing socio-demographic characteristics (see Table 2).

The focus group interview with social workers was conducted at a local welfare organization by the first author. Because of the busy work schedule of social workers, the focus group could last maximum 60 min. Therefore, only the main findings from the interviews with participants were presented to the social workers. Social workers were asked to comment on these results, focusing on results that were recognizable (to identify similarities in perspective) and results that they did not recognize (to identify differences in perspective). There was too little time to let social workers comment on all results. Therefore, the results section also contains results on which social workers did not comment. Table 3 and Table 4 present the main results for participants and social workers and also indicates on which topics the social workers did not comment.

### 2.4. Analysis

All interviews were audio recorded and the recordings were transcribed verbatim. The interviewer (first author) wrote memos after each interview and during each stage of the data analysis. Analyses started while data collection was still ongoing, making it an iterative process [20]. Analyses were done manually using Microsoft Word (Microsoft Inc., Redmond, WA, USA).

Data were analyzed thematically using the Framework Method [21]. This method consists of five stages: (1) familiarization, (2) identifying the thematic framework, (3) indexing, (4) charting, and (5) mapping and interpretation. The first three stages of data analysis were performed independently by the first author (for all transcripts) and two research assistants (for half of the transcripts each). Disagreements between coders were discussed until consensus was reached. During the familiarization stage, the coders listened to the audio recordings and read through the transcripts and memos of the first seven interviews, while making notes about recurring themes and other ideas about the data. The next stage was identifying the thematic framework. Based on open coding of the first seven interviews, an inductive approach was chosen to identify themes and categories from the open codes. During the indexing stage, the thematic framework was applied to all transcripts and supplemented with new emerging themes and categories. This stage was repeated by the second author for a set of ten randomly chosen transcripts. In the charting stage, a matrix was made for each health behavior in which the responses of the participants were summarized according to the three most mentioned barriers and facilitators for health behavior change and the five most mentioned ideas for a health promotion intervention. During the mapping and interpretation stage, the matrices were examined closely to make comparisons between categories within participants and comparisons between participants within categories. The full transcripts were regularly consulted to check the original wordings of participants and the context of their remarks. The final two stages of data analysis were performed by the first author and checked by the second author.

**Figure 1:**
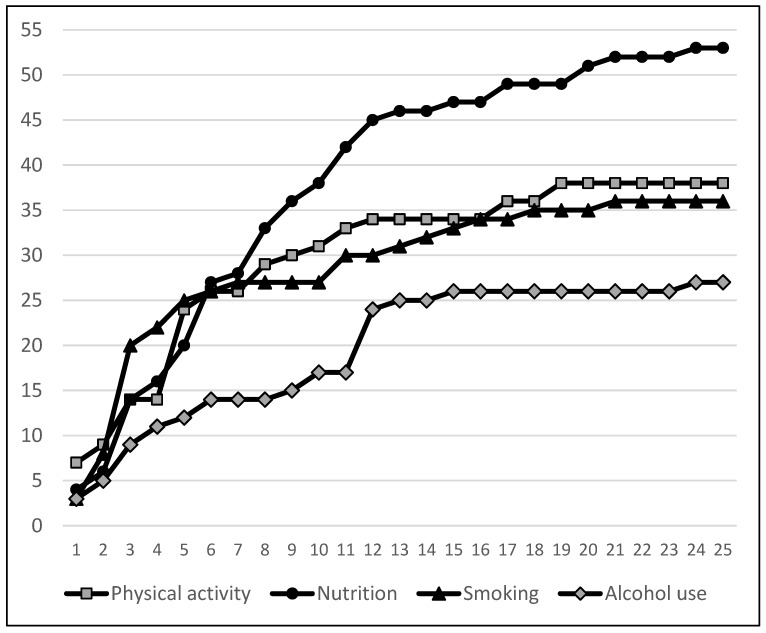
Total number of barriers and facilitators (y axis) mentioned during the 25 interviews (x axis) per health behavior.

Saturation was reached for the barriers and facilitators of physical activity after 19 interviews and for smoking after 21 interviews (see Figure 1). In interview 24, a new barrier for nutrition and a new facilitator for alcohol use was mentioned. Saturation was thus not reached for the barriers of healthy nutrition and the facilitators of reducing alcohol use.

The results section contains numbers between brackets to indicate how many respondents have given a certain answer. This is not indicated for the social workers because they were interviewed in a focus group and responses like humming and nodding were not counted.

## 3. Results

### 3.1. Participant Characteristics

As can be seen in Table 2, 11 men and 14 women participated in the interviews. Most participants had a low educational level, were unemployed, and had a low income level. Two participants were underweight, ten had a normal weight, two were overweight, and eight were obese (three participants did not report their weight).

### 3.2. Physical Activity (Participants)

Most participants (18/25) reported that they were physically active for at least half an hour each day. Most often mentioned activities were walking and cycling. A majority of participants (15/25) said they wanted to be more physically active than they were now. Some participants explained that they wanted to be more physically active, but did not have enough motivation. 

The most often mentioned barrier for more physical activity was that sports are expensive (9/25). If participants would have more money, they wanted to go to the gym (5/9) or do martial arts (3/9). “*I have a medical statement that I should do sports with the medication that I have. But the municipality doesn’t want to cooperate to help me pay that. So it is just not possible… unfortunately*” (participant #6, woman, 39 years).

Another frequently mentioned barrier for doing more physical activity (7/25) was that participants claim they cannot do more physical activity due to health issues such as vascular disease, arthrosis, or diabetes. “*That [more physical activity] is not possible right now. In February they found vascular constrictions that cannot be treated with angioplasty. (…) Overnight you lose your fitness. That’s uh… indescribable*” (participant #1, woman, 49 years). This barrier was more often mentioned by women than men.

The third most mentioned barrier for doing more physical activity (6/25) was that it takes too much time. Participants reported having too little time for physical activity because they have to take care of their children (5/6) and because they are busy with work (3/6). “*Already before we became parents, I’ve always said that I wanted to go to the gym. It’s just that I cannot find the time for that now*” (participant #15, man, 19 years). 

The most often mentioned facilitator for physical activity (10/25) was having a paid job or voluntary job that includes physically activity and/or traveling to that job on foot or by bicycle. 

Another facilitator for doing more physical activity is that people want to increase (or maintain) their feeling of fitness and want to feel more energetic (9/25). “*Of course you want to be a bit more fitter and have more energy*” (participant #14, woman, 43 years).

Many participants owned a dog (16/25) and some of them mentioned this as a facilitator for walking regularly (9/25). Most of these participants (6/9) reported walking an hour or more per day with their dog. This facilitator was more often mentioned by women than by men.

### 3.3. Physical Activity (Social Workers)

Social workers were surprised that their clients were physically active for so many minutes per day and thought this was because they counted every single minute of activity, not because they were consciously exercising.

Social workers recognized that adults from multi-problem households often say they want to be more physically active when you ask them, but they thought this to be a socially desirable response. They thought that these adults generally lack intrinsic motivation to be more physically active and make excuses. “*Because first you get ‘sports are expensive’, then I say ‘you can also go for a walk’, ‘yes, but I don’t have time for that’. (…) And then finally I say ‘but you have a free half hour after dinner, you can take the kids with you’. ‘Yes, but I also have bad knees’, because that’s the next excuse. I wonder whether they really want it*” (social worker #4). This quote illustrates that social workers recognized all above mentioned barriers of participants, but framed them as excuses instead of as valid reasons for not doing more physical activity.

Social workers acknowledged that active work or transport to work or school is a facilitator for physical activity among this target group. “*Maybe also because they don’t have money for a car, this can also work positively because they have to do everything by bike or on foot. And not having a sedentary job, doing household chores, and bringing the children to school*” (social worker #8). Additionally, social workers confirmed that many of their clients from multi-problem households own a dog and this may help with getting more physical activity, although according to the social workers, not all of them walk their dog.

### 3.4. Nutrition (Participants)

Few participants reported a healthy food pattern. Participants reported not eating vegetables daily (19/25), not eating fruit daily (16/25), drinking several sugary drinks per day (11/25), skipping meals (11/25), eating unhealthy snacks (10/25), eating high-fat bread toppings (8/25), and eating white bread (8/25). Most participants (17/25) did not want to eat healthier. Some thought that they already had a healthy dietary pattern (7/25), although they often at the same time reported regular intake of unhealthy foods and drinks. An example is a man who reported drinking two liters of soft drinks per day and ate no fruits and still considered his diet as healthy. “*Yes, I eat healthy*” (Interviewer: would you want to eat more healthy?) “*No, I think it is good already*” (participant #7, man, 42 years).

One third of participants reported receiving food from the food bank (8/25) and all of them reported this as a barrier for eating healthy. Participants reported receiving unhealthy foods from the food bank, such as white bread, chocolate, potato chips, candy, French fries, chocolate sprinkles, fat meat, and ready-made meals. They also received healthy foods from the food bank, but vegetables often had to be eaten the same day and there was little variation in the fruits and vegetables that were provided. The food bank package was not enough for the entire week and people mostly bought meat, pancakes, pizza, sugary drinks, coffee, and sugar to supplement the package. “*It depends on what you get. And you have to make do with it. And yes, whether that’s healthy or not. You have to consume something. (…) So uh we don’t have much choice. (…) But it is not a healthy way, absolutely not*” (participant #14, woman, 43 years). 

A barrier that was not mentioned by participants themselves, but was derived from their remarks about healthy nutrition was that some had incorrect knowledge about healthy nutrition (7/25). Examples of incorrect knowledge are that fruit juice and salty crackers are healthy, that artificial sweeteners are unhealthy, and that the current nutritional guidelines contain not enough nutrients. “*If you really stick to the nutritional guidelines they have now… you don’t get enough [nutrients]. My children are almost never sick. Those children that really eat according to the guidelines, they are sick all the time*” (participant #1, woman, 49 years). 

Some participants (6/25) mentioned the price of healthy foods as a barrier. Especially vegetables were mentioned as being too expensive. This barrier was most often mentioned by participants who received food bank packages. 

The most often reported factor that facilitates healthy eating is when participants (9/25) have a specific health-related reason to eat healthily. Examples are vascular disease, diabetes, high blood pressure, and high cholesterol. Most respondents with these medical conditions report to have changed their food pattern. “*Because my blood sugar was too high and the doctor said that if I kept going like this then I would have only one and a half years left. With two children growing up I thought it was time for a radical change, it really was a wake-up call*” (participant #10, man, 41 years).

Another facilitator for healthy nutrition was that some participants wanted to lose weight (6/25). Participants who reported a specific health-related reason to eat healthily were also more likely to mention that they wanted to eat healthily to lose weight. “*It is important to lose weight and I try everything to lose weight, like keeping track of what I eat, not snacking so much.. But it is not going very quickly*” (participant #20, man, 40 years).

A facilitator for healthy nutrition that was only mentioned by women (4/25) was that they wanted to take proper care of their children. They explained that they would not eat healthily for themselves, but they wanted to eat healthily for their children. “*You do have children, and a bit of vegetables is part of it. Because when you are alone then it is like... yes, what does it matter?*” (participant #18, woman, 33 years). 

### 3.5. Nutrition (Social Workers)

Social workers recognized the unhealthy food patterns of their clients and explained that there are often generational patterns of unhealthy nutrition in this group. “*I think they never learned it. If I see how they are raised… I think that no one ever took them grocery shopping*” (social worker #4).

When responding to the barriers for eating healthy among the interviewed adults from multi-problem households, some social workers explained that they were aware of the fact that the food bank package also contains unhealthy foods while others were not aware of this. Social workers also reported that there is too little knowledge about healthy nutrition among their clients. In response to the third barrier (healthy nutrition is expensive), social workers said that many households have other priorities than healthy nutrition. “*This of course depends on what they think is more important. Cigarettes are more important than that your child eats vegetables every day*” (social worker #4).

In terms of facilitators for healthy nutrition, social workers only commented on the facilitator that clients want to take proper care of their children. Social workers thought it could help to emphasize the responsibility to eat healthily for the children.

### 3.6. Smoking (Participants)

Most participants smoked (16/25) and a few had quit smoking (3/25). Of the smokers, most wanted to quit someday (9/16) but now was definitely not the time. Some participants clearly stated that they did not want to quit (4/16) and others did not know yet (3/16). Most smokers (10/16) had quit smoking in the past and succeeded for several weeks, months, or even years before they started smoking again.

Almost all (former) smokers reported that smoking helps relieve stress (16/19) and that this is a major barrier for quitting smoking. The fact that participants tended to experience a lot of stress made it difficult for them to quit smoking. A stressful situation was also the number one reason why smokers who had quit smoking reported to have started again. One woman explained that she made a very conscious choice to start smoking again to release tension: “*I had quit for five years and then the stress began… it just started choking me (…). I got more and more panicky and restless and then they wanted to up my medications. And I would have walked around like a zombie. So I was like, you know what, I’ll just take a cigarette first and uh… and yes, I regret it very much, because five years is a lot. But I am happy that I don’t have to take so much medication*” (participant #5, woman, 32 years). 

Many participants acknowledged that quitting smoking is very difficult (13/19). Some participants were surprised when they noticed that they could easily quit drinking for a while but had much more difficulty quitting smoking. Because quitting smoking is so difficult, some participants reported to have cut down on the number of cigarettes they smoked per day instead. Two women admitted that they could not even stop smoking when they were pregnant. “*When I got pregnant from my youngest daughter... I thought it was really difficult to quit smoking, and… [sigh] then I was like, I’ll only take three cigarettes in the evenings*” (participant #12, woman, 36 years). 

Another barrier that was mentioned by participants (5/19) is that many people in their social environment smoked. When smokers who had quit smoking started smoking again, this was often because they were with friends who smoked. “*I did try. I quit for half a year or a year and then on a given day you just start again. When you are in a group or in a bar. You just automatically join them*” (participant #11, man, 45 years). This barrier was more often reported by men than women.

Many (former) smokers mentioned health-related reasons to quit smoking (13/19). A specific health-related reason to quit smoking, such as vascular disease or high cholesterol, was often mentioned as the main reason to quit by those who succeeded in quitting (for a while). For those who did not have a specific health-related reason to quit, health in general was sometimes mentioned as a reason to quit. However, among these participants, it was one of several reasons that were not decisive. “*It is little things together: the costs, health, the children… lots of things... There is not something like, well, BAM, this throws you over the threshold*” (participant #3, man, 34 years).

Another facilitator for quitting smoking for many participants (9/19) was that smoking is expensive. In about half of these cases the costs are mentioned as one of many reasons to quit smoking and in the other half it is the main reason for participants. “*Actually it is solely about the money. Not because of my health, I think I have already fucked everything up anyway*” (participant #15, man, 19 years).

Finally, some participants (5/19) could not really explain why they suddenly decided to quit smoking; they just did it in the spur of the moment or because they wanted to try what it was like to quit smoking. “*Yeah, I don’t know, it was just at once, just from one day to the next.. I thought: I’m not going to smoke anymore. I woke up and I said: I am really not going to smoke. And I just persevered in one go, for two months*” (participant #4, man, 27 years).

### 3.7. Smoking (Social Workers)

Some social workers said they had an even higher percentage of smoking clients from multi-problem households than the percentage among the study participants, while others said it was about the same. Social workers recognized that their smoking clients were not ready to quit smoking.

Social workers acknowledged that their clients experience a lot of stress, but thought that they used stress as an excuse to not quit smoking. Social workers did recognize that their clients thought that quitting smoking is very difficult and that some female clients keep smoking while being pregnant. They added that they receive a lot of resistance when they try to stimulate someone to quit smoking.

Social workers thought that health reasons are not always enough motivation to quit smoking. “*I even see people with health problems of whom I really know.. that the general practitioner (…) has said ‘you shouldn’t do it’ and afterwards I still see them smoking*” (social worker #1). They did not comment on other facilitators for quitting smoking.

### 3.8. Alcohol Use (Participants)

Most participants did not drink alcohol at all (8/25) or only at very special occasions such as New Year’s Eve (8/25). Some participants drank one glass or less per day (6/25), two participants regularly participated in binge drinking in the weekends or at parties, and one participant drank two to four glasses of alcohol per day. Only two participants reported that they may want to reduce their alcohol intake one day. 

Most participants did not drink much or, when they did, they did not perceive it as too much. Therefore, they also did not report barriers for reducing or stopping alcohol use. However, certain beliefs about alcohol use could be considered as barriers. A belief that was mentioned a lot by women was that drinking was seen as sociable when they were with friends or relatives (7/25). “*It’s not that I need it or anything, but it is just when there is a sociable evening I just take it*” (respondent #16, woman, 19 years). 

Another belief that was mentioned about alcohol by participants was that it tastes good and is relaxing (6/25). People enjoy drinking during dinner, when watching television in the evening, or when they are at a party. “*It gives a certain relaxation. And I think it tastes good with dinner*” (participant #24, man, 59 years).

The most often mentioned reason (9/25) for not drinking much alcohol or no alcohol at all is that participants had alcoholics in their social environment, and this caused them to have negative associations with alcohol. This was more often mentioned by women than by men. The women who mentioned this as reason reported being violated or abused by their father or ex-husband who was an alcoholic. “*My ex-husband drank too much and he got uhm.. (…) abused me.. and aggressive. We’re talking about sixteen half liters per evening so I have difficulty with people who start drinking too much beer. Two beers is fine by me and if it is more, I start uh.. to get tense*” (participant #5, woman, 32 years). 

Some participants (5/25) reported that drinking is not something for them. They do not like the taste or the effect of alcohol. This was more often reported by participants who also reported the previous facilitator. “*I just don’t have anything with alcohol. No, not a thing*” (participant #6, female, 39 years).

Other participants (5/25) reported that it is not difficult to drink less. When people wanted to drink less this was because of a variety of reasons, such as being pregnant, because they noticed they drank too much, because of medications, because their partner asked them to, or because they did not have the money. None of the participants reported any difficulty with reducing or quitting alcohol use. “*When I just met my wife, I drank 3 to 4, sometimes even 6 half liters per evening (…) and then she said one day ‘can you reduce it somewhat?’, and I was like ‘sure, I can reduce it’. (…) If she feels better if I do then uh.. that’s fine by me. If she is happy, then I am happy. And I am not addicted to it*” (participant #20, man, 40 years). This was more often mentioned by men than by women. 

### 3.9. Alcohol Use (Social Workers)

The low levels of alcohol use among the study participants was a topic of discussion among the social workers. One social worker reported that her clients with problematic alcohol use that she invited to participate did not want to participate in an interview about health behavior. However, other social workers said that they did not think that problematic alcohol use was something that would be more prevalent among multi-problem households than among the general population.

Based on the barrier that drinking is sociable, social workers commented that alcohol use is considered acceptable in our society. Social workers recognized the pattern of not drinking (much) alcohol due to a history of violence or abuse by the alcoholic fathers or ex-husbands of their female clients.

Social workers explained that they recognized from most of their clients that they do not have difficulty with reducing their alcohol consumption, but that they also had clients who were problem drinkers and did have difficulty with reducing their alcohol intake on their own.

### 3.10. Health Promotion Intervention (Participants)

The most often mentioned ideas from participants about a health promotion intervention are summarized in Table 4. The most mentioned idea (12/25) was to organize something (such as a neighborhood party or barbecue) where one can meet other people. A participant explained that having more social contacts can be a reason to stay healthy. “*That [to have social contacts] is important. Also to stay healthy. Why would you eat less and why would you reduce alcohol if you have no social contacts?*” (participant #17, man, 52 years). Another participant said that the intervention should not be promoted as an intervention about health, but as one about social contacts. “*Nobody will come a program about health and welfare. (…) Neighborhood contact! Focusing more on that and then sneaking the welfare and health into it*” (participant #8, woman, 39 years).

Another often mentioned idea was to organize an outdoor sports or playing event (10/25). Six participants mentioned this as something for children, but where adults could also go and meet other people. “*When there is a recurring event that would be nice, for example a sports and outdoor playing event. Once a month for example, where you can go and then meet other people*” (participant #5, woman, 32 years).

Several participants were enthusiastic about cooking and eating together (6/25). Two participants mentioned explicitly that the financial barrier should be taken into account. “*Cooking together, eating, also doing groceries together. Then you also take into account the financials*” (participant #16, woman, 19 years).

A walking group was mentioned by a couple of participants (4/25). “It’s totally free and I think there would be more people who would have time for that during the day (…) I would like that very much, to do that together” (participant #25, woman, 43 years).

The same amount of participants (4/25) mentioned that children should do more things that does not include smartphones, tablets, computers, or television. The importance of physical activity for children was stressed and the importance of being outside in nature without electronics. “*If you look at them now. They are sitting next to each other on the couch using their phones. There is no activity anymore. They are not doing anything anymore*” (participant #13, man, 58 years).

### 3.11. Health Promotion Intervention (Social Workers)

There was limited time to discuss participants’ ideas for a health promotion intervention with the social workers. Social workers recognized the importance of social contacts and organizing activities in groups where clients can meet other people. “*Sport groups or something (…). Then you also stimulate social contacts, that they get to know other people*” (social worker #6). They agreed that it can help to organize something for children, which may stimulate the adults too. “*Parents are very motivated to give the good example to their children. I think that if you focus on children, that parents then want to come along too*” (social worker #4). All of them were enthusiastic about the idea of cooking and eating together and someone suggested to frame it as a budget cooking class. Some social workers thought a walking group was a good idea, while others did not think that people from multi-problem households would actually show up. Social workers did not comment on the last intervention idea of participants.

**Table 3:** Behavior, intention, top three barriers, and top three facilitators per health behavior according to adults from multi-problem households and responses of social workers.

		Adults from Multi-Problem Households	Social Workers
Physical activity	Behavior	Most (18) ^†^ were physically active for at least half an hour each day	Surprised, thought this was because they counted every single minute
	Intention	Majority (15) said they wanted to be more physically active	Thought this to be a socially desirable response
	Barriers	Sports are expensive (9)	Recognized this as an often used excuse
		Health restrictions for doing more physical activity (7)	Recognized this as an often used excuse
		Physical activity takes too much time (6)	Recognized this as an often used excuse
	Facilitators	Having physically active work or physically active transport to work (10)	Recognized this
		Desire to improve their fitness (9)	Did not comment on this
		Walking regularly because of dog ownership (9)	Recognized this
Nutrition	Behavior	Participants reported not eating vegetables daily (19), not eating fruit daily (16), drinking several sugary drinks per day (11), skipping meals (11), eating unhealthy snacks (10), eating high-fat bread toppings (8), and eating white bread (8)	Recognized this and explained that there are often generational patterns of unhealthy nutrition in this group
	Intention	Most (17) did not want to eat healthier	Did not comment on this
	Barriers	Receiving food from the food bank (8)	Some were aware that this is a barrier
		Incorrect knowledge about healthy nutrition (7)	Recognized this
		Healthy nutrition is expensive (6)	Recognized this
	Facilitators	Health-related reasons to eat healthily (9)	Did not comment on this
		Eating healthily to lose weight (6)	Did not comment on this
		Wanting to care for their children with healthy nutrition (4)	Recognized this
Smoking	Behavior	Most participant (16) smoked and a few had quit smoking (3)	Some had a higher percentage of smoking clients, while with others it was the same
	Intention	Of the smokers, most wanted to quit someday but now was definitely not the time (9)	Recognized this
	Barriers	Smoking helps relieve stress (16)	Acknowledged that clients have a lot of stress, but thought they used it as an excuse to not quit smoking
		Quitting smoking is difficult (12)	Recognized this and received resistance when trying to stimulate quitting
		Many people in their social environment smoke (5)	Did not comment on this
	Facilitators	Health-related reasons to quit smoking (13)	Commented that health reasons are not always enough to be able to quit smoking
		Smoking is expensive (9)	Did not comment on this
		Suddenly decided to quit smoking (5)	Did not comment on this
Alcohol use	Behavior	Most participants did not drink alcohol at all (8) or only at very special occasions (8)	Said that alcoholics did not participate in the interview (selection effect), but problematic alcohol use is not particularly prevalent among these households
	Intention	Only two participants wanted to reduce their alcohol intake (2)	Did not comment on this
	Barriers	Drinking alcohol is sociable (7)	Recognized that drinking is considered acceptable
		Drinking alcohol tastes good and is relaxing (6)	Did not comment on this
	Facilitators	Resistance to drink alcohol because of alcoholics in social environment (9)	Recognized this among female clients
		Drinking is not for me (5)	Did not comment on this
		It is not difficult to drink less alcohol (5)	Recognized this from most of their clients, but some did have difficulties to drink less

^†^ Numbers between brackets are the number of participants who have mentioned this. This is not indicated for the social workers because they were interviewed in a focus group and responses like humming and nodding were not counted.

**Table 4:** Most often mentioned ideas for a health promotion intervention by participants and responses of social workers.

Adults from Multi-Problem Households	Social Workers
Something social (12) ^†^	Recognized the importance of social contacts
Outdoor sports or playing event (10)	Agreed that it can help to organize something for children, which may stimulate adults too
Cook and eat together (6)	Were enthusiastic about this idea and suggested to frame it as a budget cooking class
Walking group (4)	Some thought this was a good idea, while others did not think that their clients would show up
Children’s activities in nature (4)	Did not comment on this

^†^ Numbers between brackets are the number of participants who have mentioned this. This is not indicated for the social workers because they were interviewed in a focus group and responses like humming and nodding were not counted.

## 4. Discussion

The aim of the current study was to identify barriers and facilitators for health behavior change among adults from multi-problem households (referred to as ‘participants’) in the Netherlands, as well as to identify ideas for program development. Another goal of this study was to examine similarities and differences in perspectives of adults from multi-problem households and social workers who work with this group.

Mentioned facilitators for physical activity were having physically active work or transport to work, desire to improve their fitness, and walking regularly with their dog. The main reason for not doing sports was that this is too expensive. This is in line with previous studies that have shown that costs are the main reported barrier for physical activity among unemployed and low-income adults [22,23]. When looking at the mentioned facilitators and barriers for physical activity from a theoretical point of view, the data suggests that mostly perceived barriers -reducing self-efficacy- and actual barriers are mentioned, factors that are also identified in models such as the Theory of Planned Behavior [24] and the I-Change Model [25]. However, social workers differed in their perceptions of physical activity among adults from multi-problem households and framed their barriers as ‘excuses’. It therefore remains to be seen whether physical activity increases if this target group gets more time and money.

If participants had medical conditions such as diabetes or high blood pressure, this motivated them to eat healthy, but others were often not motivated to change their diet. All participants who received a food bank package reported this as a barrier for healthy eating. Previous research from the Netherlands has indeed found that food bank packages do not meet nutritional guidelines [26]. Ensuring healthier food bank packages would help the recipients to make healthier choices. Additionally, incorrect knowledge about healthy nutrition should be corrected. Social workers noted that there are often generational patterns of unhealthy nutrition because nobody learned their clients how to prepare healthy meals. This is in line with previous studies among low socio-economic status groups [8,14]. From a theoretical point of view, these factors suggest that there are actual or perceived barriers for healthy nutrition and that level of awareness (knowledge and risk perceptions) plays a role [25]. Social workers also recognized these factors and thus seemed to have similar perspectives about nutrition among multi-problem households.

Smokers and former smokers reported that smoking helps relieve stress and that this is a major barrier for quitting smoking. Social workers acknowledged that their clients experience a lot of stress, but thought that they used stress as an ‘excuse’ to not quit smoking. Previous studies have shown that stress is more often reported as barrier for quitting smoking among low socioeconomic groups than among high socioeconomic groups [9,12,14]. Other barriers were related to self-efficacy and social influences. The most mentioned facilitator was that participants had health-related reasons to quit smoking, relating to their attitude concerning health according to the I-Change Model [25]. Although this was mentioned a lot among participants, social workers did not agree that health-related reasons were enough for their clients to actually quit smoking.

The most frequent mentioned barriers and facilitators for reducing or stopping alcohol use were social factors and attitudes. A number of participants reported bad experiences with alcoholics, such as violence and abuse. Previous studies have shown that more alcohol consumption is associated with increased risk of family violence and that this relationship is stronger among low socioeconomic groups [27,28]. One social worker explained that her alcoholic clients did not want to participate in an interview. If this is the case, then our results may be heavily influenced by selection bias. Other social workers said that problematic alcohol use is not something that is more prevalent among their clients than among the general population. This is contrary to previous studies on problematic alcohol use among low socio-economic status groups and those living in stressful conditions [2,7,11]. It may be explained by the fact that in the municipality of Apeldoorn problematic alcohol users are mostly treated in addiction treatment and not by social workers. Either way, the barriers and facilitators for reducing alcohol intake that we found in our study are likely not generalizable.

Participants mentioned several intervention ideas for a health promotion intervention for multi-problem households. They suggested to organize something social, to organize an outdoor sports or playing event, to cook and eat together, to start a walking group, and to have activities for children that include physical activity in nature without smartphones and other electronic equipment. Previous studies have shown that a good social network is important for increasing self-rated health [29] and for the effectiveness of care for multi-problem households [1,30]. Cooking classes for school-aged children have positive effects on food preferences, attitudes, and behavior [31]. Cooking classes for adults also show positive effects, although there is little research of high quality [32]. Organizing walking groups can be successful [33,34,35] and it combines an activity that is free with a social component [36]. Additionally, walking is something many already do and that is feasible for persons with obesity [37]. The wish of the participants to reduce children’s screen time can be met with existing effective interventions [38]. Effective interventions include information provision about reducing screen time, setting screen-time goals, and restricting access to electronic equipment. It is notable that the ideas from participants for the health promotion intervention included mostly ideas about physical activity, one idea about nutrition, and one idea about social network enhancement, but not about tobacco or alcohol reduction. Social workers were mostly enthusiastic about the intervention ideas of their clients. The only thing where some social workers were hesitant about was whether clients would actually show up for a walking group. Indeed, attrition from walking groups seems to be larger among low socioeconomic groups [39]. Social interaction and walking with dogs may help to decrease attrition among this group.

Our study was performed among adults from multi-problem households with a low socioeconomic status, but results are not different for other groups with a low socio-economic status. As mentioned in the introduction, psychological distress [9,10,12,13,14], a lack of knowledge about health risks and about healthy behaviors [8,14], having social networks of friends and family with unhealthy lifestyles [9,12,14], and the costs of sports and healthy nutrition [8,9,10,14,15] are barriers among low socioeconomic groups in general. Something that is unique about our target group is that they receive intensive family home care by social workers. This can be seen as an opportunity, because these social workers could potentially help them with changing their health behavior. Efforts are needed to ensure that social workers realize the importance of health behavior change and get allocated sufficient time to not only help families with their most pressing problems but to also stimulate health behavior change.

## 5. Limitations

A limitation of our study was that saturation was not reached for the barriers of healthy nutrition and the facilitators of reducing alcohol use, because a new barrier for nutrition and a new facilitator for alcohol use was mentioned in interview 24. However, in the three interviews before that, no new barriers or facilitators were mentioned for nutrition and alcohol use. Three interviews without new ideas emerging is sometimes used as a stopping criterion for saturation in qualitative interviews [40]. Additionally, our results were compared with results from a focus group interview with social workers who worked with multi-problem households, which made full saturation among participants somewhat less important.

Another limitation was that the situation in which the interview took place was not the same for each participant. Interviews were conducted at participants’ homes. During eight of the interviews it was not possible to conduct the interview with no one else present in the same room (e.g., children, partner, or social worker). The answers that participants gave may have been influenced by the other people who were present.

It was very difficult for most respondents to estimate their monthly gross household income. Most respondents got weekly money from the municipal debt assistance and were not aware of their actual income. Although there was a ‘don’t know’ option and a ‘refusal’ option, participants wanted to answer each question and sometimes chose the middle income category when they did not know the answer.

Finally, this study does not give sufficient information yet about which health promotion interventions would be effective and acceptable for multi-problem households. Participants were only asked for ideas that they had themselves and they did not all comment on all mentioned ideas. Additionally, interviews lasted on average only 31 minutes while barriers, facilitators, and intervention ideas concerning four health behaviors were discussed. Therefore, further formative research is needed among this target group. We recommend to conduct interviews on only one or two health behaviors, which leaves more time to discuss them thoroughly. We also recommend to conduct follow-up interviews (or focus groups interviews) in which mentioned intervention ideas are discussed with all participants. And, finally, we recommend to co-create interventions together with all relevant stakeholders.

## 6. Conclusions

Adults from multi-problem households experience many barriers for health behavior change, such as financial barriers, incorrect knowledge, and high levels of stress. Facilitators for health behavior change among these group include having physically active (transport to) work, motivation to improve fitness and to lose weight, and for some a specific medical condition that they have. These insights into barriers and facilitators for health behavior change among adults from multi-problem households offer preliminary directions for intervention development. Participants also mentioned several ideas for a health promotion intervention themselves. The most mentioned ideas from participants were to organize something social, to organize an outdoor sports or playing event, to cook and eat together, to start a walking group, and to have activities for children that include physical activity in nature without smartphones and other electronic equipment. Social workers agreed that it is useful to organize social activities, to organize something for the children which could also attract their parents, and to organize cooking classes. Besides these activities that are directed towards the target group, it may be important to influence their environment by educating social workers and ensuring healthier food bank packages.

## Figures and Tables

**Table 1:** Interview guide.

Physical activity	What kind of physical activity do you perform? How many minutes are you physically active in a regular week? Do you want to do more physical activity? If yes: What kinds of physical activity? Why do you want that? Why haven’t you done so already? What makes it hard? If no: Why not? Did you used to do more physical activity? If yes: What kinds of physical activity? What helped you at that time? What has changed now? Have you tried to be more physically active in the past? And were you successful? If tried and succeeded: What kinds of physical activity? What helped you at that time? If tried and failed: What kinds of physical activity? What helped you at that time? Why didn’t you succeed?
Nutrition	Do you get a food bank package? What do you eat and drink on a regular day? Do you want to eat and drink more healthy? If yes: Why do you want that? Why haven’t you done so already? What makes it hard? If no: Why not? Did you used to eat and drink more healthy? If yes: What helped you at that time? What has changed now? Have you tried to eat and drink more healthy in the past? And were you successful? If tried and succeeded: What helped you at that time? If tried and failed: What helped you at that time? Why didn’t you succeed?
Smoking	Do you smoke tobacco? Do you want to quit smoking? If yes: Why do you want that? Why haven’t you done so already? What makes it hard? If no: Why not? Have you tried to quit smoking in the past? And were you successful? If tried and succeeded: What helped you at that time? If tried and failed: What helped you at that time? Why didn’t you succeed?
Alcohol use	How much alcohol do you drink in a regular week? Do you want to reduce your alcohol intake? If yes: Why do you want that? Why haven’t you done so already? What makes it hard? If no: Why not? Do you used to drink less alcohol? If yes: What helped you at that time? What has changed now? Have you tried to reduce your alcohol intake in the past? And were you successful? If tried and succeeded: What helped you at that time? If tried and failed: What helped you at that time? Why didn’t you succeed?
Health promotion intervention	We want to develop a program about healthy living for people who received social care. Such a program would promote physical activity, healthy nutrition, or smoking cessation, or would reduce alcohol intake. Would you have ideas about what such an intervention could consist of? What would be fun and useful to do?

**Table 2:** Participant characteristics.

	N (%)
Gender	
Male	11 (44)
Female	14 (56)
Age (M, SD)	40.4 (11.9)
Age of children (M, SD)	13.2 (8.8)
Household composition	
One adult living alone	6 (24)
One adult living with child(ren)	8 (32)
Two adults living without children	1 (4)
Two adults living with child(ren) from them as a couple	7 (28)
Two adults living with child(ren) from previous relationship(s)	3 (12)
Highest level of completed education	
Primary education	5 (20)
Lower pre-vocational secondary education	11 (44)
Middle pre-vocational secondary education	4 (16)
Secondary vocational education	2 (8)
Senior general secondary education and pre-university education	3 (12)
Higher professional education and university bachelor or master	0 (0)
Employment	
Paid employment	5 (20)
Unpaid employment	5 (20)
No employment	15 (60)
Number of hours of paid employment per week (M, SD)	32.9 (21.0)
Monthly gross household income	
Less than €1000 per month	9 (36)
Between €1000 and €1500 per month	7 (28)
Between €1500 and €2000 per month	6 (24)
Between €2000 and €2500 per month	3 (12)
More than €2500 per month	0 (0)
Perceived income adequacy (managing with household income)	
Very difficult	3 (12)
Fairly difficult	7 (28)
Moderately difficult	6 (24)
Moderately easy	3 (12)
Fairly easy	5 (20)
Very easy	1 (4)
Body Mass Index (M, SD)	
<18.5 (underweight)	2 (8)
18.5–25 (normal weight)	10 (40)
25–30 (overweight)	2 (8)
30+ (obese)	8 (32)
Not reported	3 (12)
